# On Kephalosis

**Published:** 1839-01

**Authors:** 


Art. II.—*A Fact in the Natural Children, hitherto unobserved, which explains much concerning Infantile Diseases and Mortality.* By John Gardner, Surgeon.—*London,* 1838. 8vo. pp. 23.

Mr. Gardner's little pamphlet of twenty-three pages contains some clear and well-expressed remarks on the changes which are observed during the gradual progress of development up to the adult age. He observes, that the whole body "becomes thirty times as great as at birth, whilst the weight of the brain is only quadrupled; and of this increase the greater part is accomplished within six years, by which time it has tripled its original weight." (p. 7.) This shows that the most considerable, and therefore important, changes of development which the brain undergoes are confined to a comparatively short period of time; a period which must therefore be more peculiarly liable to those derangements which result from a loss of the due order and regularity with which the successive steps and changes of growth are effected.

In a vast metropolis like this, where so many causes almost unavoidably conspire to check that tone and vigour of health upon which the normal action of the different functions and other vital processes depend, it appears to us extraordinary that the changes of development and growth belonging to early life, and which require such great efforts of the system to effect, can ever be completed, in addition to the other and more common changes, without overturning the equilibrium of the nicely balanced machine, and deranging the regularity and harmony of its numerous but beautifully adapted movements.

"Corresponding with the rate of its (the brain's) growth," says Mr. Gardner, "is the development or assumption of its powers and offices. The instinctive actions of sucking and crying, the vital impulses to the organs of nutrition, circulation, and secretion, being necessary to existence, the brain is adapted to their exercise. The sensations of vision, taste, smell, hearing, and touch, the muscular states, and the power of controlling by the will the muscular motions, come gradually into play. The preparation of the future mind proceeds in order; certain associations are indissolubly established between the different sensations, before the power of recording impressions or the consciousness of passing feelings comes into action. In due time, recollection, memory, the faculty of speech, the power of walking, and other less obvious functions, become successively developed; all requiring the gradual and orderly growth of the brain, until its completion in size marks the attainment of all its faculties, which require but due culture and exercise to achieve all the mighty workings of mind." (p. 7.)

Mr. Gardner brings his subject before the reader under three heads or propositions:

"1st. The gradual, equal, or rather normal, development of the brain is essentially necessary to a state of average health and *vital* strength in the constitution of childhood.

"2d. An irregular, unequal, accelerated, *abnormal* (or disordered) *rate of growth of the brain*, or any part of this organ, gives a peculiar character to a child's constitution, rendering it vitally weak, susceptible of morbid impressions, and an easy prey to ordinary diseases.

"3d. Such a condition of the brain is widely prevalent among children, and is *traceable in the external form of the head.*" (p. 9.)

This condition of the brain the author proposes to designate by the term *Kephalosis.*

"The signs by which this state of the brain is recognized being manifested in the external form of the head, although with certain concomitant marks in the general system." (p. 11.)

"These irregularities in its form, even when slight, will be easily detected by a practised eye; but, when the development has proceeded to an important extent, a mere casual observer will perceive it." . . . "The overhanging of some point of the fore part of the brain beyond the lowest point,—the situation of which is marked by the root of the nose,—is always an indication that the brain is undergoing an abnormal development." . . . "The lowest point of the anterior lobes of the brain is always most forward in a condition of perfect health; and the line of the forehead makes either a right angle or a slightly acute angle with it. If that line form an obtuse angle at this point, kephalosis exists; and the forms of the bones, their connexion, and relation to the fontanelles are such as to lead most commonly to this deviation from the healthy outline of the forehead, at whatever part of the brain the accelerated growth is proceeding." (p. 13.)

Mr. Gardner considers that, by carefully observing the shape of the head in children, the practitioner may frequently detect any tendency to morbid development of the brain, and divert the mischief which is threatened. He attempts to distinguish this condition from rachitis and struma, but, we think, without success; both of these diseases being essentially connected with, or even dependent upon, a defective state of the processes of development. Mr. G. concludes with a few observations on the signs and consequences of Kephalosis. In these we see nothing particularly new or interesting, nor in any way equal to the passages which we have just quoted. He appears to have read Dr. Gooch's translation of Gölis's work on Hydrocephalus with much attention; but the essay would have been much more complete if we had been favoured with even a glimpse of the author's method of treatment. When the symptoms and characters of a disease are described, which the author tells us he can cure, we naturally look for some account of the means by which he effects this desirable end. The observations at the commencement are too good to make us suppose that any intentional concealment is intended; and we therefore trust that Mr. Gardner will, ere long, favour us with a fuller consideration of so important a subject, of which the present pamphlet is merely the preamble.

